# The developing human brain: age‐related changes in cortical, subcortical, and cerebellar anatomy

**DOI:** 10.1002/brb3.457

**Published:** 2016-03-22

**Authors:** Dafna Sussman, Rachel C. Leung, M. Mallar Chakravarty, Jason P. Lerch, Margot J. Taylor

**Affiliations:** ^1^ Physiology and Experimental MedicineThe Hospital for Sick ChildrenUniversity of Toronto555 University AvenueTorontoOntarioM5G 1X8Canada; ^2^ Diagnostic Imaging ResearchThe Hospital for Sick ChildrenUniversity of Toronto555 University AvenueTorontoOntarioM5G 1X8Canada; ^3^ Cerebral Imaging CentreDouglas Mental Health University InstituteVerdunQuebecCanada; ^4^ Departments of Psychiatry and Biomedical EngineeringMcGill UniversityMontrealQuebecCanada; ^5^ Mouse Imaging Centre (MICe)The Hospital for Sick Children25 Orde StreetTorontoOntarioM5T 3H7Canada; ^6^ Department of Medical BiophysicsUniversity of Toronto101 College StreetTorontoOntarioM5G 1L7Canada

**Keywords:** Basal ganglia volume, cerebellar anatomy, cortical anatomy, hippocampal anatomy, magnetic resonance imaging, pediatric neuroanatomical development, typically developing children

## Abstract

**Introduction:**

This study is the first to characterize normal development and sex differences across neuroanatomical structures in cortical, subcortical, and cerebellar brain regions in a single large cohort.

**Methods:**

One hundred and ninety‐two magnetic resonance images were examined from 96 typically developing females and 96 age‐matched typically developing males from 4 to 18 years of age. Image segmentation of the cortex was conducted with CIVET, while that of the cerebellum, hippocampi, thalamus, and basal ganglia were conducted using the MAGeT algorithm.

**Results:**

Cortical thickness analysis revealed that most cortical regions decrease linearly, while surface area increases linearly with age. Volume relative to total cerebrum followed a quadratic trend with age, with only the left supramarginal gyrus showing sexual dimorphism. Hippocampal relative volume increased linearly, while the thalamus, caudate, and putamen decreased linearly, and the cerebellum did not change with age. The relative volumes of several subcortical subregions followed inverted U‐shaped trends that peaked at ~12 years of age. Many subcortical structures were found to be larger in females than in males, independently of age, while others showed a sex‐by‐age interaction.

**Conclusion:**

This study provides a comprehensive assessment of cortical, subcortical, and cerebellar growth patterns during normal development, and draws attention to the role of sex on neuroanatomical maturation throughout childhood and adolescence.

## Introduction

Brain development begins very early in prenatal life, continues into young adulthood, and is accompanied by dramatic changes in gray and white matter (Jetha and Segalowitz 2012). While a variety of developmental analyses have been carried out with the goal of understanding the progressive and regressive neuroanatomical changes, no study has investigated the cortical, subcortical, and cerebellar anatomy in a single large cohort with equal numbers of male and female children and adolescents. Characterization of co‐occurring anatomical changes and associated sexual dimorphism that take place during the first two decades of life can aid in understanding typical and atypical developmental patterns and sex differences in neurodevelopmental disorders.

Previous studies on total cerebral volume found that it followed an inverted U‐shaped trajectory. Namely, it increased very rapidly in infancy, peaked around 10–12 year of age, and then declined during late adolescence (Lenroot et al. 2007). Total cerebral gray matter was found to follow a similar trend (Sowell et al. 2001; Gogtay et al. 2004; Lenroot et al. 2007). Contrary to these observations, white matter volume was found to follow a steady and almost linear increase throughout childhood and adolescence (Pfefferbaum et al. 1994; Giedd et al. 1999; Lenroot et al. 2007). Understanding of sexually dimorphic patterns of brain development may shed light on underlying mechanisms responsible for sex differences, symptomatology, and age of onset of a variety of pediatric neuropsychiatric disorders (Giedd et al. 1999).

Investigations of developmental patterns of cortical structures are also available in the literature. Sowell et al. (2004) investigated cortical thickness in children aged 5–11 years and found significant cortical thinning over broad areas of right frontal and bilateral parieto‐occipital regions (Sowell et al. 2004). Cortical thickening, on the other hand, was restricted to the left inferior frontal and bilateral posterior temporal regions, which are associated with speech and language abilities (Toga et al. 2006). Using data from 3‐ to 40‐year‐old participants, Shaw et al. (2008) found that the thickness of most of the lateral frontal, lateral temporal, and parieto‐occipital cortices followed cubic age‐related trajectories. Only the insula and anterior cingulate followed quadratic trajectories, while the posterior orbitofrontal and frontal operculum regions primarily followed linear trajectories.

Other studies have analyzed the hippocampi and basal ganglia structures (Gogtay et al. 2004; Dennison et al. 2013; Goddings et al. 2014) and showed volumetric changes that interacted both with age and sex, depending on the structure. For example, the caudate, putamen, and thalamus had a greater decline in volume in adolescent females than males (12–18 years of age) (Dennison et al. 2013).

Only few studies have been conducted on both cortical and subcortical development in the same subjects. Sowell et al. (2002) included thirty‐five 7‐ to 16‐year‐old typically developing participants (Sowell et al. 2002), and found an age‐related increase in brain volume, accompanied by a proportional increase in white matter volume. There was also a relative increase in volume in the mesial temporal cortex, caudate, thalamus, and basomesial diencephalic structures in females compared with males. More recently, Muftuler et al. (2011) completed a similar study on 126 children but only aged 6–10 years (Muftuler et al. 2011). They observed age‐related cortical thinning, along with a relative volume increase in the thalamus. The only structure to show sex differences was the right insula, which showed a slight increase in thickness with age in girls.

Analyses of cerebellar development during childhood and adolescence appear to be even sparser in the literature. One of the first studies was conducted by Tiemeier et al. (2010), who showed that total cerebellar volume in 5‐ to 24‐year‐old participants followed an inverted U‐shaped trajectory that peaked at around 12 years in females and 16 years in males (Tiemeier et al. 2010). Developmental trajectories of cerebellar subdivisions were also investigated and those of the inferior posterior lobe were found to peak earlier than those of superior posterior and anterior lobes, which are thought to have a more recent phylogenetic origin (Tiemeier et al. 2010). Developmental changes in myelinated and unmyelinated cerebellar volumes were later also investigated in 50 boys and girls between 3 months and 12.7 years of age (Wu et al. 2011). This study showed that cerebellar volume increases logarithmically, with an initial rapid increase within the first 2 years. This rapid increase was found to occur earlier in females, particularly in the case of total cerebellar and unmyelinated volumes (Wu et al. 2011). Other studies of cerebellar growth have focused on specific developmental disorders or insults (Bauer et al. 2009; Bolduc et al. 2012), on the effect of gestational age (Goldstein et al. 2002), or on a narrower age range (Choe et al. 2013).

Our study provides a comprehensive cross‐sectional analysis of the age‐related brain changes that occur during childhood and adolescence. The study utilizes magnetic resonance imaging (MRI) collected from female and male participants aged 4–18 years to construct the developmental trajectories of cortical, subcortical, as well as cerebellar structures. To conduct the cerebellar and subcortical analysis, a new and accurate segmentation method called MAGeT (multiple automatically generated templates from a single labeled brain) is employed (Winterburn et al. 2013). It delineates not only subcortical regions, but also subregions within them. In our analysis, the effect of sex is investigated to elucidate the extent to which sexual dimorphism impacts each neuroanatomical region.

## Materials and Methods

This study was approved by The Hospital for Sick Children Research Ethics Board and conducted in accordance with its guidelines. Informed written consent was obtained from all participants and/or their parents.

### Participants

Two hundred sixty‐one typically developing male and female participants between the ages of 4 and 18 were recruited from the local community as controls in various neurofunctional and neurostructural studies. Participants were excluded if they were born premature, had a history of psychiatric illness and/or learning disability, were taking psychotropic medications during the study, presented with any contraindication to MRI, or had gross neurostructural abnormalities or significant artifacts in their MRI scan. IQ was assessed in participants through the Wechsler Abbreviated Scale of Intelligence (WASI). Nearest neighbor matching of remaining male and female participants was then carried out using propensity score matching technique (Ho et al. 2011), which calculates the probability of a subject being selected to participate given their age (i.e., their observed covariate). This matching imitates randomization by creating a sample of female subjects comparable on observed covariate (age) to a sample of male subjects. One‐to‐one matching of male‐to‐female participants was conducted, yielding a smaller group of 96 males and 96 females (192 in total) for which the age covariate value was the closest. This group of matched participants was then analyzed for statistical differences in selected metrics.

### MRI acquisition

Image acquisition was conducted on a 3T Siemens Trio MRI scanner (MAGNETOM Tim Trio, Siemens AG, Erlangen, Germany) with a 12‐channel head coil. A T1‐weighted 3D sagittal magnetization‐prepared rapid gradient echo (MP‐RAGE) sequence was used with TR/TE/TI/FA = 2300 msec/2.96 msec/900 msec/9 degrees. This sequence, which was carried out on all participants, provided 1 mm isotropic voxels, a FOV of 192 × 240 × 256 mm with a 256 × 256 matrix, and 192 slices. During the scan, participants were allowed to watch a movie of their choice using MR‐compatible goggles and earphones; head motion was restricted using foam padding.

### Image processing

#### Cortical analysis

All MR images were registered into a common three‐dimensional space and corrected for RF inhomogeneity artifacts using the corticometric iterative vertex‐based estimation of thickness (CIVET) pipeline (Collins et al. 1995; Sled et al. 1998; Ad‐Dab'bagh et al. 2006). This pipeline uses discrete tag point classification followed by partial volume information to classify the different tissues as gray matter, white matter, and cerebrospinal fluid (Zijdenbos et al. 2002; Kim et al. 2005). The Constrained Laplacian Anatomical Segmentation using Proximities (CLASP) method is then utilized to produce the gray and white matter surfaces (Kim et al. 2005) which, in turn, are used in computing the cortical surface area (SA). To improve the accuracy of surface identification, white matter surfaces are expanded until they reach the gray matter or cerebrospinal fluid surface boundary (Kim et al. 2005). This creates four surfaces, two per hemisphere, each containing 40,962 vertices, which are registered to the MNI ICBM152 surface template that allows for a groupwise statistical comparison. The distance between surface boundaries is used in computing the cortical thickness (CT) which, together with SA, is utilized in computing the cortical volume (CV) (Ad‐Dab'bagh et al. 2006). CT was analyzed using both a lobe‐based analysis of the 78 neuroanatomical structures segmented in the AAL atlas, as well as with a vertex‐based analysis of all 81,924 vertices (Tzourio‐Mazoyer et al. 2002).

#### Cerebellar and subcortical analysis of the hippocampus, striatum, palladium, and thalamus

The basal ganglia, cerebellum, and hippocampi in all MR images were segmented using MAGeT algorithm (Chakravarty et al. 2013), whose improved accuracy compared with other similar techniques was recently demonstrated (Park et al. 2014). This algorithm uses one accurately and manually segmented atlas for the basal ganglia structures and five accurately and manually segmented atlases for the cerebellum and the hippocampi (Chakravarty et al. 2006; Winterburn et al. 2013; Park et al. 2014). An arbitrary subset of MR images is designated as “templates,” for which multiple anatomical segmentations are generated through pairwise registration with each of the atlases. This yields a template library of labeled candidate segmentations for each structure. Each subject brain is then registered to these segmentations. The resulting labeled voxels undergo a “voxel voting” procedure, during which only the most frequently occurring segmentation label at each voxel is retained, resulting in the most accurate final anatomical segmentation for a particular structure (Collins and Pruessner 2010). These segmentations are then used in calculating the volume of each structure, which is utilized in a relative volume calculation for each subregion. Cerebellar subregions' volumes were divided by the total cerebellar volume. For all other cerebrum structures, the volumes were divided by the total cerebrum volume, as computed through CIVET's volumetric sum of the gray matter (GM), white matter (WM), and cerebrospinal fluid.

Each MAGeT atlas further segmented subdivisions within each anatomical structure of interest. The basal ganglia atlas divided the structure into the thalamus, globus pallidus, caudate, and putamen. The cerebellar atlas divided the structure into 13 regions: lobules I–II, lobule III, lobule IV, lobule V, lobule VI, Crus I, Crus II, lobule VIIb, lobule VIIIa, lobule VIIIb, lobule IX, lobule X, white matter. Finally, the hippocampal atlas divided each hippocampus into the following five subfields: CA1, subiculum, CA4/dentate gyrus, CA2/3, stratum radiatum/lacunosum/moleculare. Subfields in the right and left hemisphere were combined and the total was used in the data analyses.

### Statistical analyses

All statistical analyses were conducted in R. The dependence of each imaging metric: CT, CV, SA, cerebellar, and subcortical volumes on each of the variables: sex, age, as well as their interaction terms were tested using an analysis of variance (ANOVA). A similar analysis was conducted by replacing age by age range. Two age ranges were considered in the analysis: 4–11 and 12–18, approximately corresponding to childhood and adolescent periods. The effect of total brain volume on WM and GM was also tested. A natural spline with varying degrees of freedom between one and three was applied to age. The three fits (linear, quadratic, and cubic) were then compared with an ANOVA and the one that yielded a significant improvement in fit (*P* < 0.05) was used in the statistical test of that imaging metric.

Correction for multiple comparisons was carried out through the false discovery rate (FDR) technique (Benjamini and Yekutieli 2001), which was applied to each term of the ANOVA in all imaging metric analyses, as well as in the vertex‐based cortical thickness analysis. Only FDR values equal to or less than 10% were considered significant. In those cases where significance was reported, the exact FDR values are also included with the results. Bar graphs show the mean along with the 95% confidence interval.

## Results

### Demographic parameters

Information about the number and IQ of male and female participants is summarized in Table 1.

**Table 1:** Number and IQ of male and female participants per age range

Age range	Age (mean ± SD)	Males		Females	
		*N*	IQ (mean ± SD)	*N*	IQ (mean ± SD)
4–7	6.2 ± 1.3	24	111.7 ± 15.1	24	109.7 ± 17.9
8–11	10.2 ± 1.2	14	115.6 ± 17.3	19	114.9 ± 13.5
12–15	14.4 ± 1.1	40	109.2 ± 10.2	38	109.0 ± 11.5
16–18	16.8 ± 0.6	18	116.6 ± 10.4	15	111.1 ± 12.3
Total	12.0 ± 4.1	96	115.7 ± 9.8	96	110.5 ± 12.4

IQ, intelligence quotient; SD, standard deviation.

### Cortical parameters

**Figure 1:**
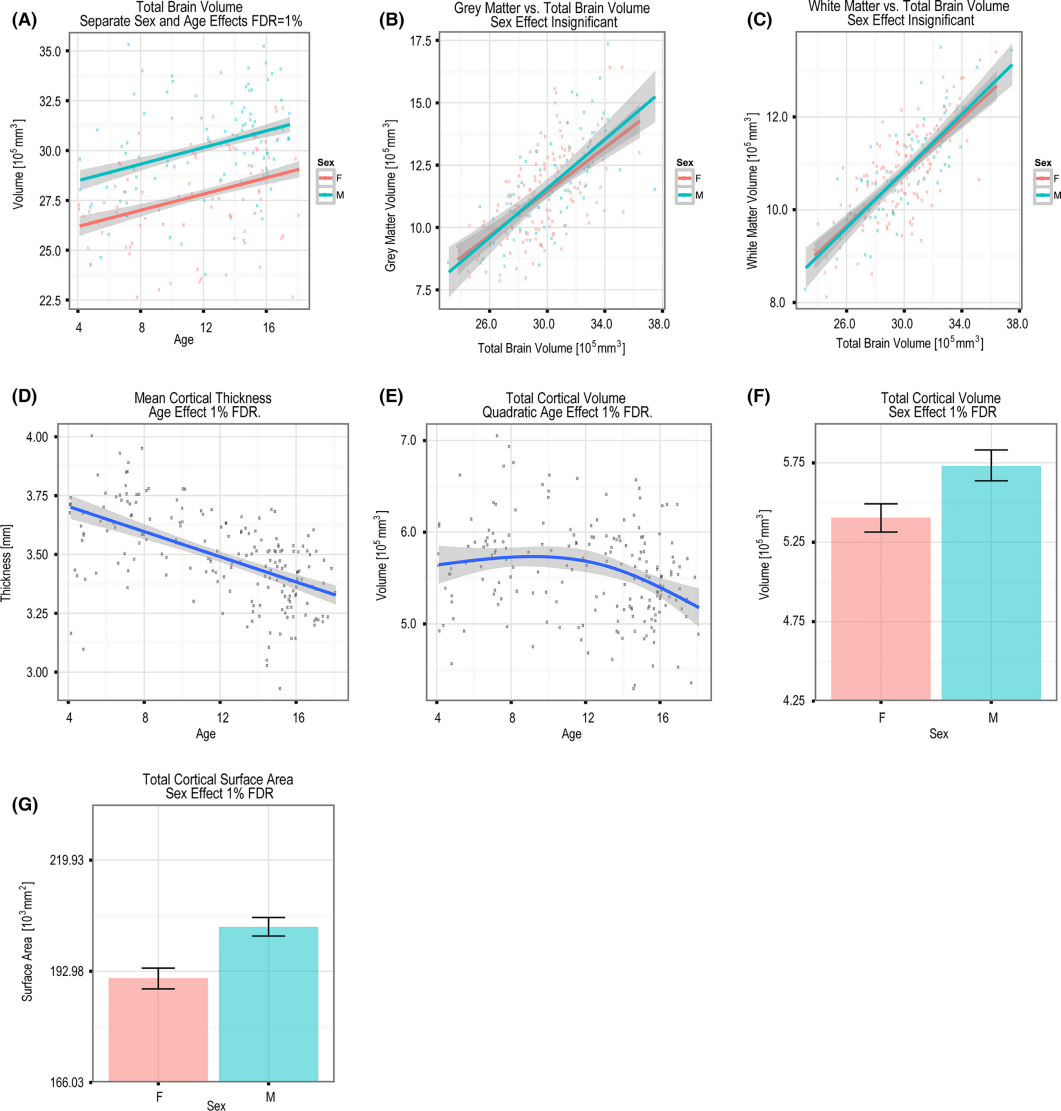
(A) Separate sex and age effects on whole brain volume. The effect of total brain volume on (B) gray matter and (C) white matter volume. Age effect on (D) mean CT and (E) total CV. Sex effect on (F) total CV and (G) total SA (FDR = 1%). These effects disappeared when CV and SA were corrected for total cerebral volume.

Total brain volume was found to increase linearly with age (Fig. 1A). Both gray matter and white matter volumes increased linearly with increasing total brain volume (Fig. 1B and C) and mean CT (Fig. 1D) decreased linearly with age (FDR = 1%). Total brain, GM, and WM volumes were all larger in males compared with females by 7.3%, 10.4%, and 7.5%, respectively, when computed across age. Total CV followed a quadratic fit that started decreasing at around 12 years of age (Fig. 1E). Both SA and CV were significantly larger in males than females (Fig. 1F and G; FDR = 1%); however, these effects become insignificant when SA and CV were corrected for total brain volume. Neither SA nor CV showed a significant age‐by‐sex interaction.

**Figure 2:**
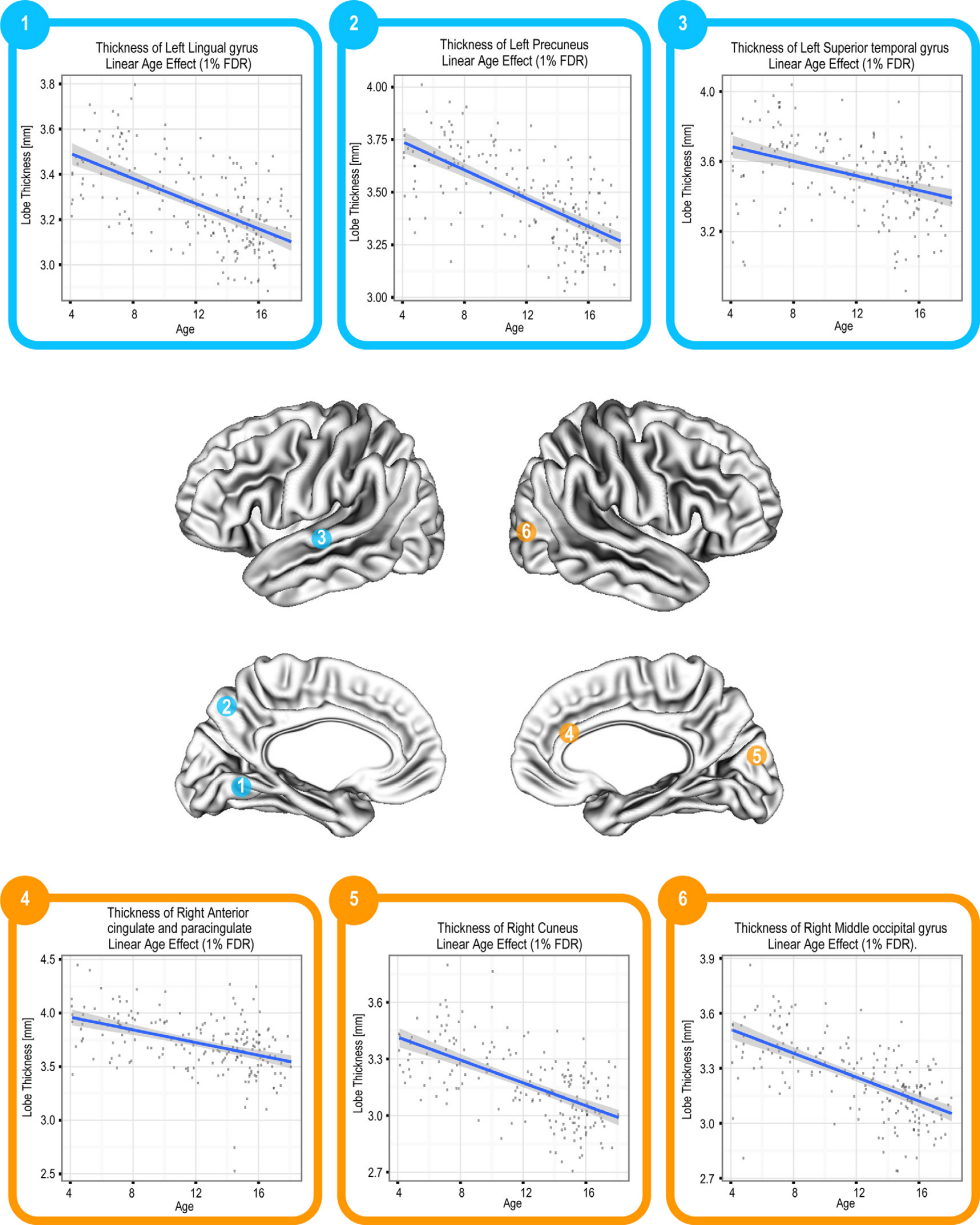
Linear decrease in CT in a range of regions (FDR = 1%).

**Figure 3:**
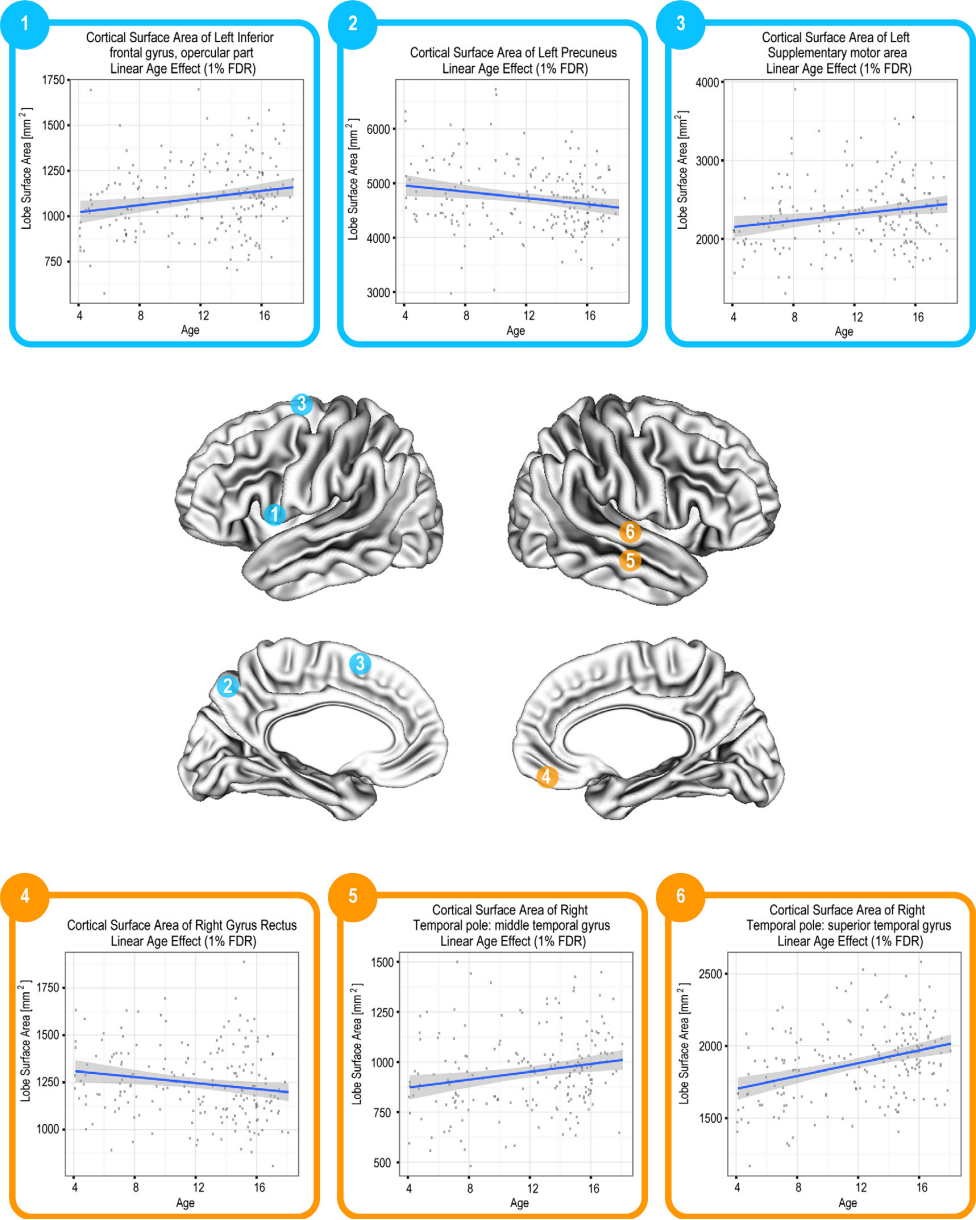
Linear change in SA in a number of different regions (FDR = 1–10%).

CT was found to decrease linearly with age (FDR = 1%) in all cortical regions with the exception of the bilateral superior and middle temporal poles (Fig. 2). The SA of the following cortical regions was found to vary linearly with age (FDR ≤ 10%): left inferior frontal gyrus (opercular and triangular), left supplementary motor area, left anterior cingulate and paracingulate gyrus, left inferior temporal gyrus, left parahippocampal gyrus, left precuneus, right superior temporal pole, right gyrus rectus, bilateral middle temporal pole. With the exception of the left precuneus and the right gyrus rectus, all these regions had an increasing SA with age. SA trajectories of selected regions are shown in Figure 3.

**Figure 4:**
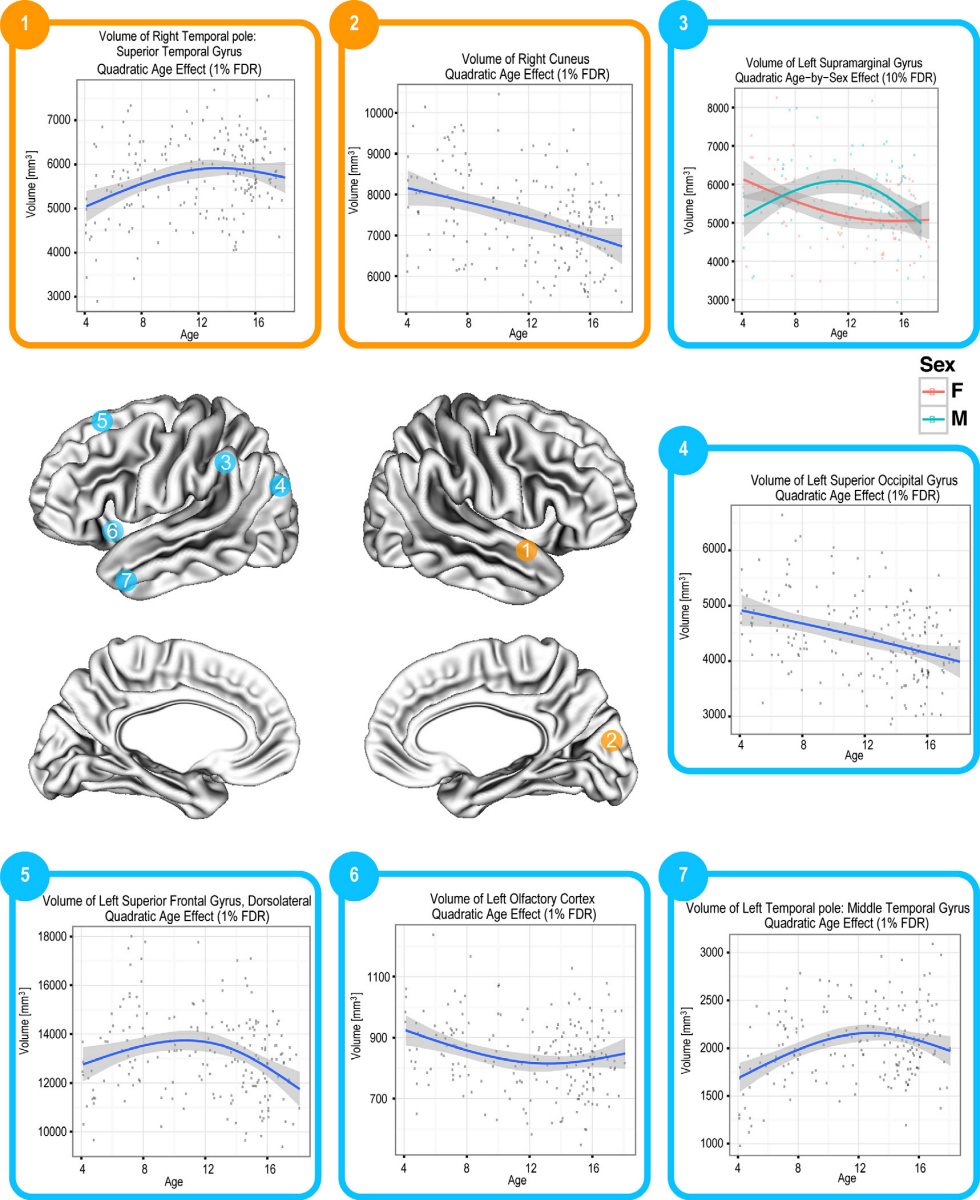
Quadratic change in CV in several different regions (FDR = 1%).

Most cortical regions were also found to change in CV as a function of age (FDR = 1%), as depicted in Figure 4. The age‐related trend in CV of each cortical region is summarized in Table 3.

**Figure 5:**
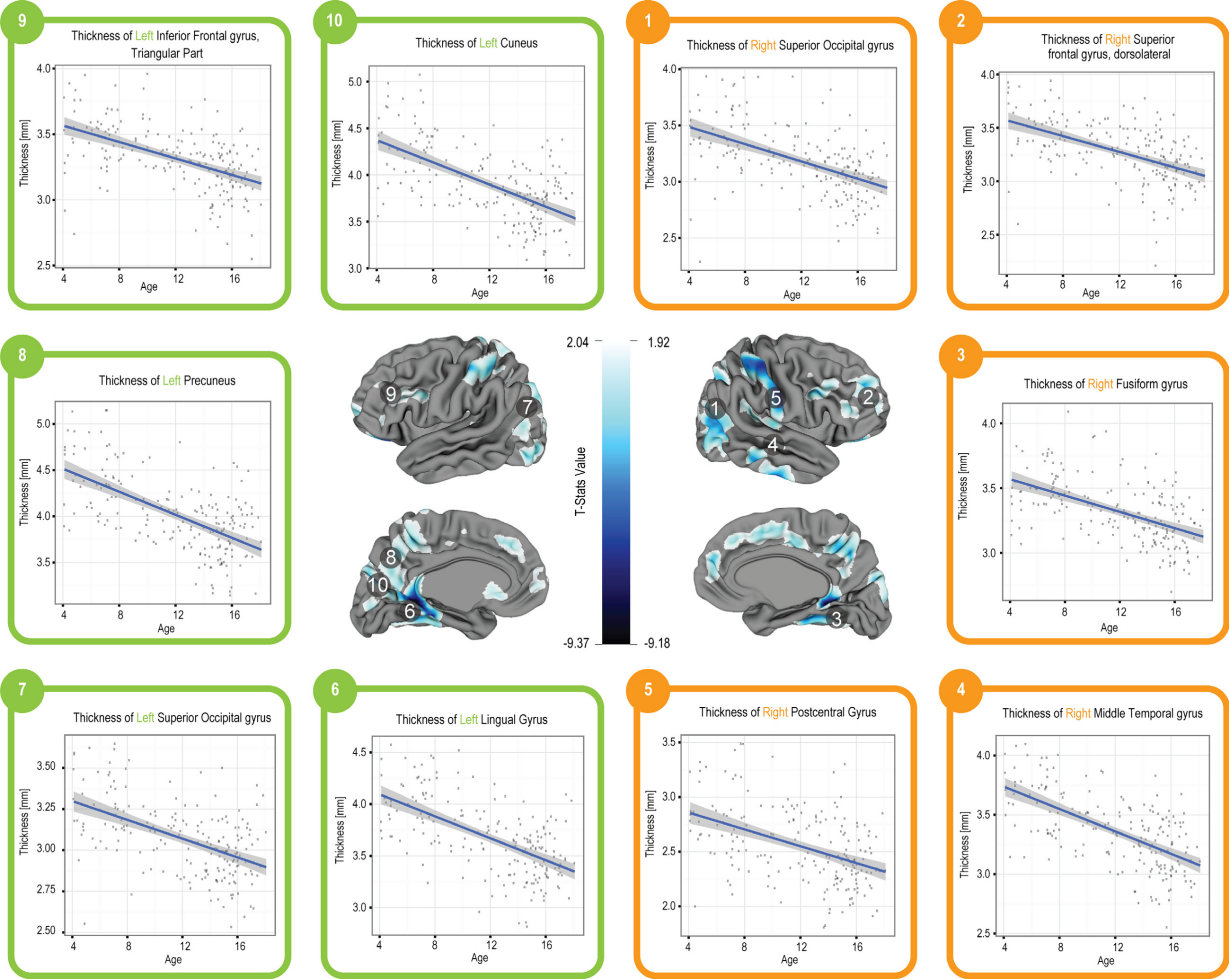
T‐statistics maps for age effect on CT using a vertex‐based analysis. Age is centered at 12 years. Blue regions indicate areas with extreme t‐stats values.

**Figure 6:**
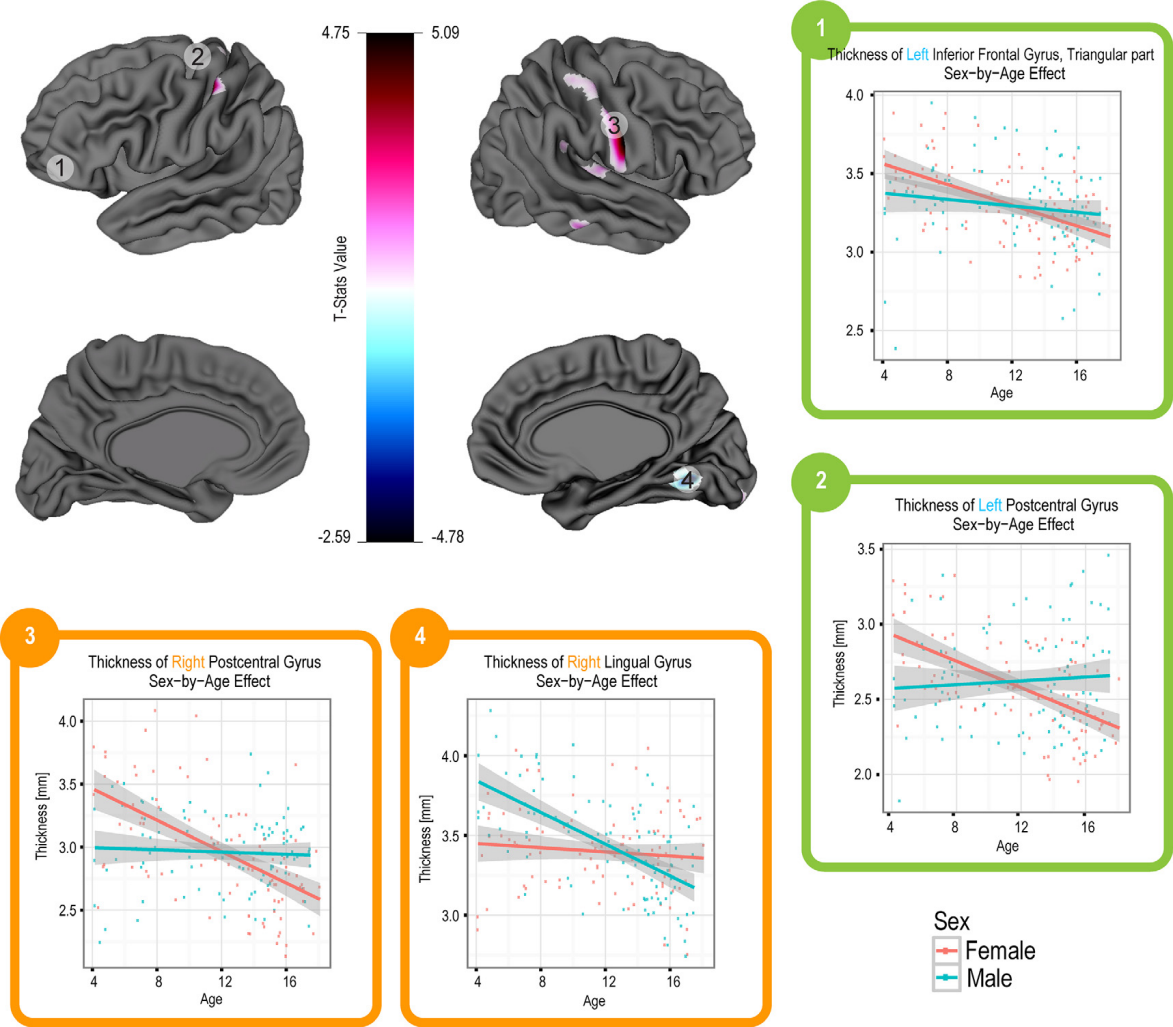
T‐statistics maps for sex‐by‐age interaction effect on CT using a vertex‐based analysis. Age is centered at 12 years. Red and blue regions indicate areas with extreme t‐stats values.

The vertex‐based analysis also showed significant age effects as well as sex‐by‐age interaction effects on CT. These results are summarized in Figures 5 and 6 which show the corresponding t‐stats maps in which age is centered at 12 years. These maps are accompanied by thickness versus age graphs of selected regions.

### Subcortical volumes

#### Hippocampi

**Figure 7:**
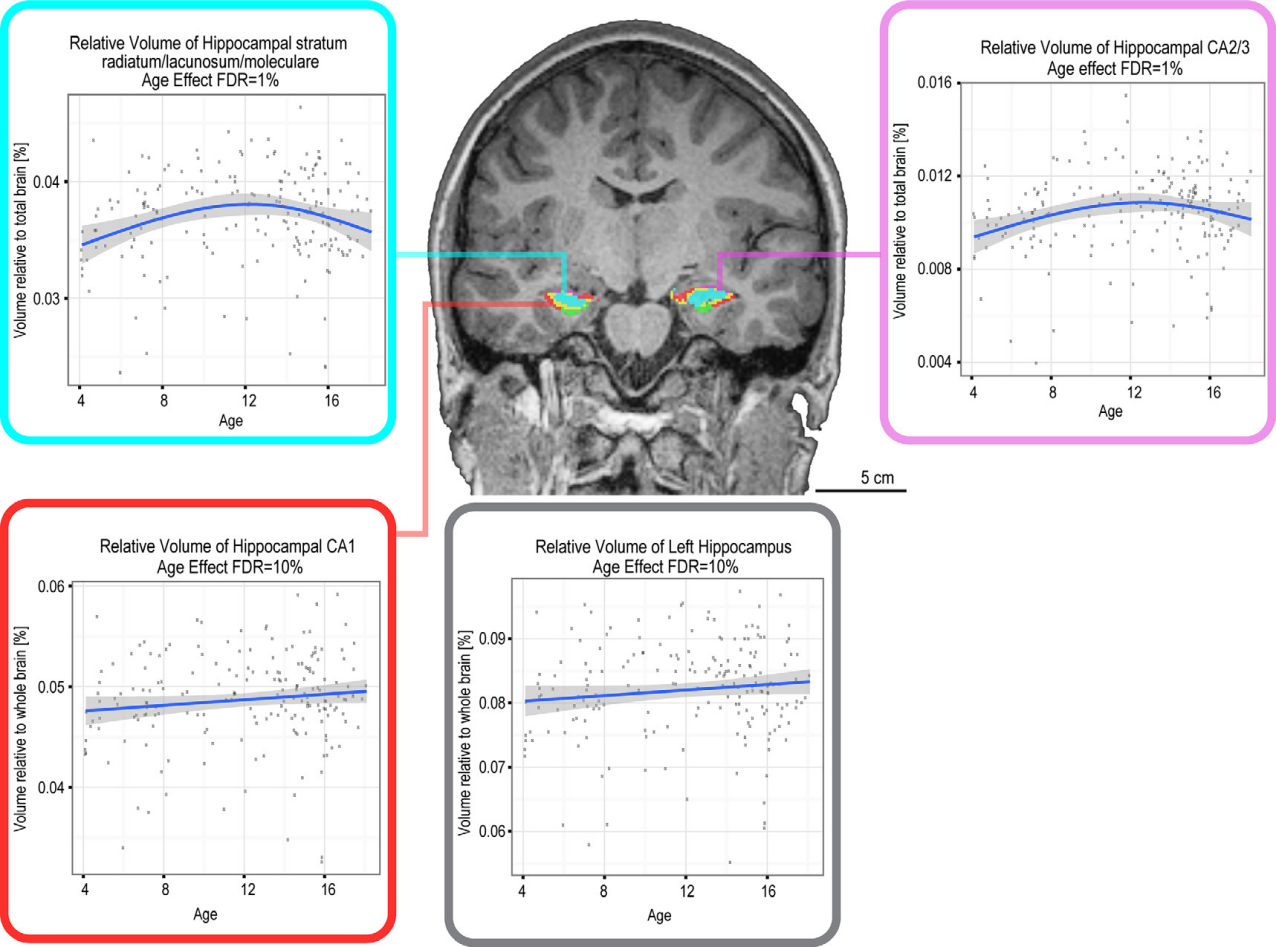
Age effect on relative volume of hippocampal subfields.

A relation with age was observed in the case of the relative volume of the left hippocampus (FDR = 10%), which increased linearly with age. A similar linear increase in relative volume was noted in hippocampal region CA1. Regions CA2–3 and the stratum radiatum also changed with age (FDR = 1%), but were best described by a quadratic fit. These results are summarized in Figure 7. Age did not affect the relative volume of the right hippocampus, the combined hippocampal volume, or any other hippocampal subfield (FDR ≥ 15%).

Independently of age, the relative volume of each hippocampus, CA4/dentate gyrus, CA2‐3, and the stratum radiatum were all found to be significantly larger in the females than the males (FDR = 1–5%), as summarized in Table 2. Sex‐by‐age interaction was not found in any hippocampal lobe or subfield.

**Table 2:** Hippocampal, basal ganglia, and cerebellar structures whose relative volumes significantly differ (*P* < 0.05, corrected) by sex (mean ± SD)

Cortical structure	Males	Females
	Relative volume (×10^−3^, %)	Relative volume (×10^−3^, %)
F > M		
Right hippocampus	80.7 ± 7.1	83.0 ± 7.3
Left hippocampus	80.5 ± 8.0	83.5 ± 6.8
CA4/Dentate gyrus	43.9 ± 4.3	46.3 ± 3.7
CA 2–3	10.0 ± 1.9	10.9 ± 1.5
Stratum radiatum	36.4 ± 4.1	37.6 ± 3.8
Thalamus	450.4 ± 28.5	457.0 ± 26.7
Caudate	263.3 ± 31.6	271.7 ± 22.8
Cerebellar white matter	12,413.7 ± 881.3	12,642.0 ± 870.4
M > F		
Cerebellar lobule I–II	153.5 ± 27.1	145.3 ± 26.4
Cerebellar lobule III	1692.2 ± 154.1	1632.5 ± 161.6
Cerebellar lobule IV	3514.4 ± 361.9	3401.9 ± 349.9
Cerebellar lobule V	6687.0 ± 506.8	6545.5 ± 459.2

SD, standard deviation.

#### Thalamus and basal ganglia

**Figure 8:**
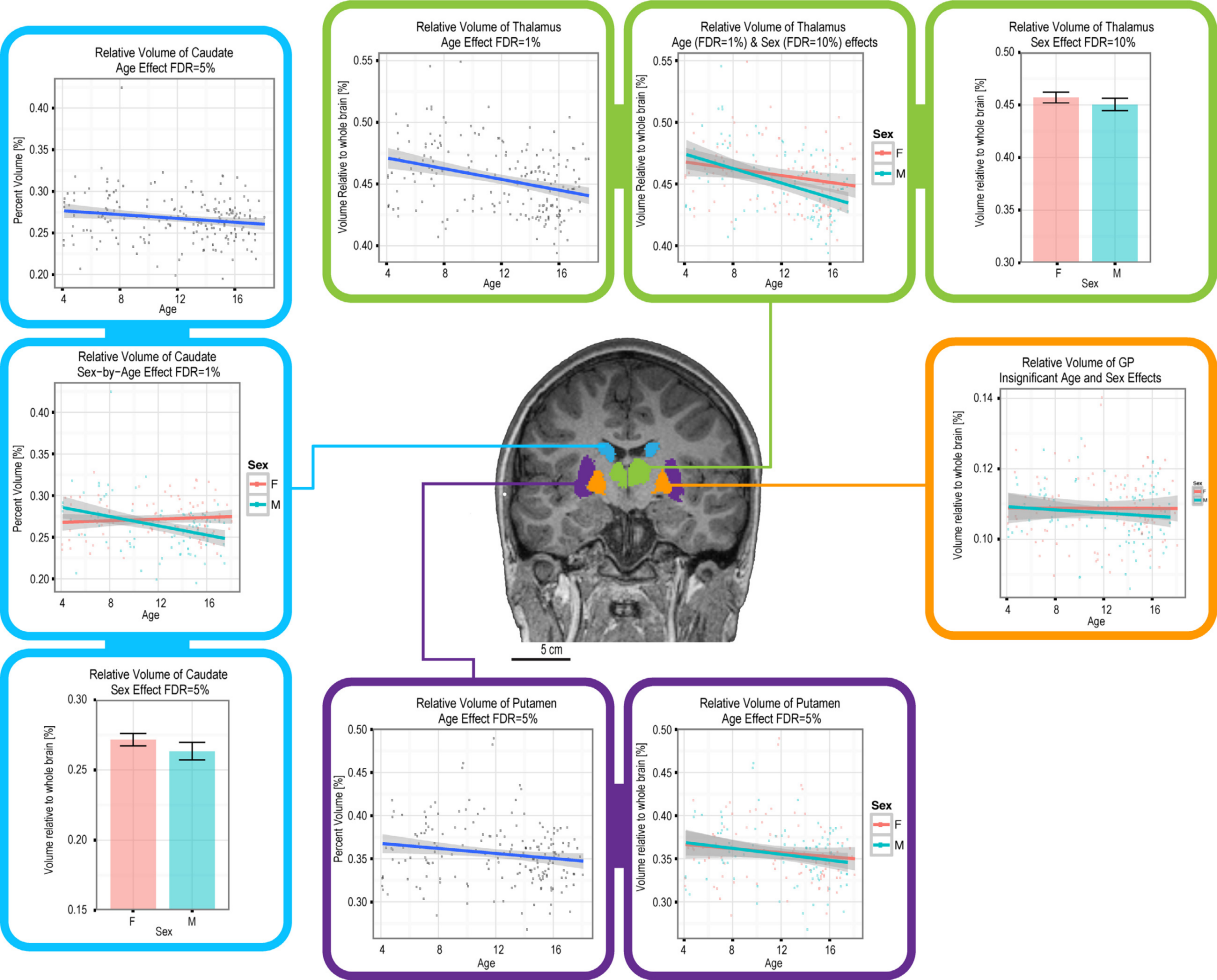
Age, sex, and sex‐by‐age interaction effects on relative volume of the thalamus, GP, putamen, and caudate.

The relative volumes of the thalamus (FDR = 1%), caudate (FDR = 5%), and putamen (FDR = 5%) all decreased with age (Fig. 8A–C). This age‐related trend also significantly interacted with sex in the case of the thalamus and caudate (Fig. 8D and E).

Independently of age, sex was also found to alter the relative volumes of the thalamus and caudate (Fig. 8F and G; Table 2). Neither age nor sex affected the relative volume of the globus pallidus.

### Cerebellum

**Figure 9:**
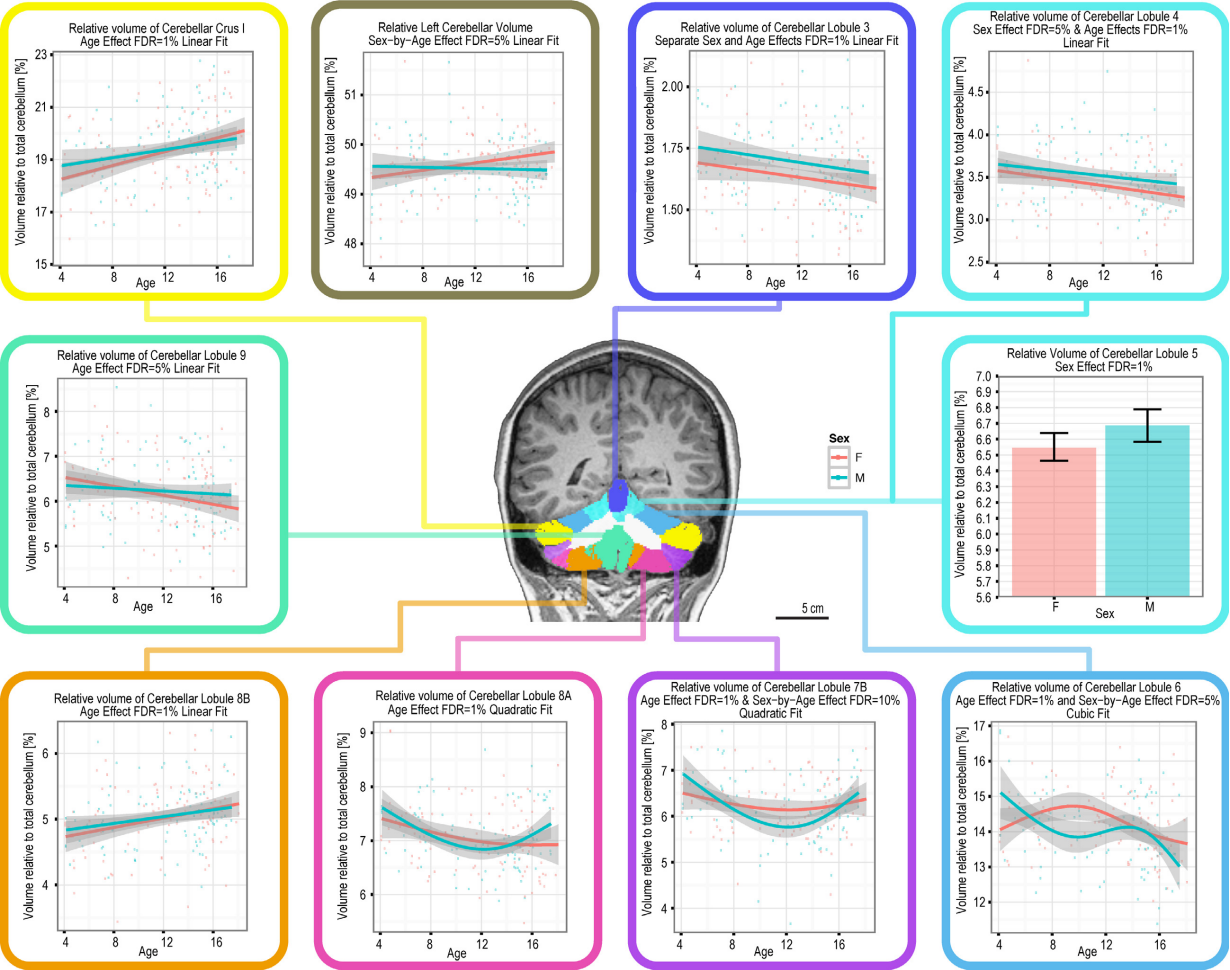
Age effect on relative volume of cerebellar lobules.

An analysis of cerebellar relative volume revealed an age effect that followed a linear trend in the case of lobules III, IV, V, VIIIB, IX, and Crus I, and a quadratic trend in the case of lobule VI, VIIB, and VIIIA. A linear sex‐by‐age interaction was found for the relative volume of the left cerebellum, which was smaller in females in childhood, until 10 years of age, and larger thereafter compared with males. A quadratic sex‐by‐age interaction was found for lobule VIIB, revealing a more drastic decrease in relative volume in males at 12 years of age. A cubic sex‐by‐age interaction was found significant for lobule VI, revealing a larger relative volume in early childhood in boys compared with girls, followed by a decline in late childhood and adolescence. Contrary to this, the relative volume of lobule VI of the female participants gradually increased from early to late childhood (10 years of age), followed by a gradual decrease in adolescence. These results are summarized in Figure 9.

The total cerebellum was found to be significantly smaller in females (117,856 ± 10,479 mm^3^) compared to males (125,607 ± 12,473 mm^3^, FDR = 1%), independently of age. Similar sex differences were noted for the relative volumes of lobules I–II, III, IV, and V, which were larger in males compared with females. The opposite trend was found for cerebellar white matter, which was larger in females than in males. These data are summarized in Table 2.

## Discussion

Both progressive and regressive neuroanatomical changes occur during childhood and adolescent development. Accurate characterization of these changes and determination of any sexual dimorphism can help understand typical developmental trajectories, as well as help in identifying neurodevelopmental disorders. This article is the first to provide a comprehensive analysis of total brain volume, gray and white matter volumes, cortical surface area (SA), cortical thickness (CT), cortical volume (CV), volume of the hippocampi, cerebellum, and basal ganglia, as well as their substructures in a single cohort of male and female participants, 4–18 years. Total brain and gray matter volumes were found to increase linearly with age at roughly the same rate in boys and girls (brain volume: *P* = 0.89, gray matter: *P* = 0.16). They were consistently larger by about 7.3% and 10.4%, respectively, in males compared with females throughout childhood and adolescence. Previous studies have reported a very similar volumetric difference of 8–10% (Giedd et al. 1997; Lenroot et al. 2007; Paus 2010); the small discrepancy may be attributed to methodological differences and sample size.

Mean CT decreased with age in a linear fashion independently of sex. A similar trend was found for most cortical regions in both the lobe‐based and voxel‐based analyses, with the exception of the bilateral middle and superior temporal gyri, which did not significantly change with age. While cortical thinning is often attributed to synaptic pruning, it may also be caused by increasing white matter maturation and the resulting change in voxel classification on the cortical white/gray matter interface, as explained by Alemán‐Gómez et al. (2013). Our results of the widespread cortical thinning are consistent with previous studies (Sowell et al. 2004; Paus et al. 2008; Muftuler et al. 2011). However, in one of these studies, regions associated with language development, the inferior frontal and left superior temporal gyri, were found to thicken between 5 and 11 years of age (Sowell et al. 2004). Since in our study we used a single linear fit for a larger age range (4–18 years), we were unable to detect transient cortical thickness changes in early childhood. Also, our use of a larger sample size and different image processing techniques may have contributed to this discrepancy. Some studies analyzing cortical thickness trajectories found nonlinear trends, such as the one by Marsh et al. (2008). Our observation of strictly linear trends might be due to a lower statistical power compared with those studies and/or the use of an age range that did not extend to older adulthood.

Similarly to CT, SA also followed a linear trend. However, while CT decreased with age, SA increased in almost all regions, with the exception of the left precuneus and right gyrus rectus. The inherent difference explaining these age‐related changes in CT and SA pivots on the phylogenetic origin of SA and CT. CT is believed to be related to the number of cells within each cortical phylogenetic column, whereas SA is dictated by the number of such columns (Rakic 1995). An expansion—or enlargement—of these columns, which is the opposite of what occurs during aging in older adults (Hogstrom et al. 2013), would then result in an increase in SA, in turn explaining the trend we are seeing during development in most cortical regions. It is unclear what causes the reduction in SA in the left precuneus and right gyrus rectus. While it could be caused by flattening of the cortex during adolescence (Alemán‐Gómez et al. 2013), it may also be due to the regional variability in SA (Chen et al. 2012), which is not accurately captured by a lobar division (Hogstrom et al. 2013). A vertex‐based SA analysis may be better suited in this case and could help distinguish between regional variability and cortical flattening.

The developmental changes of CV across age followed either a linear or a quadratic trend, depending on the cortical region. Generally, these trajectories are dictated by the relative contribution of SA and CT (Ecker et al. 2014). While studies on older adults report that CV is more closely correlated with SA than with CT (Winkler et al. 2010), in our study we do not see the same trend. The CV, SA, and CT trends are summarized in Table 3 for illustration. This discrepancy may be due to the use of a narrower age range that excluded adulthood and, thus, did not depict the cortical changes occurring later in life.

**Table 3:** Age‐related trajectories in cortical thickness (CT), surface area (SA), and volume (CV)

Cortical structure	CT	SA	CV
Left precentral gyrus	↘	–	CD
Right precentral gyrus	↘	–	↘
Left superior frontal gyrus, dorsolateral	↘	–	CD
Right superior frontal gyrus, dorsolateral	↘	–	CD
Left superior frontal gyrus, orbital part	↘	–	↘
Right superior frontal gyrus, orbital part	↘	–	↘
Left middle frontal gyrus	↘	–	CD
Right middle frontal gyrus	↘	–	↘
Left middle frontal gyrus orbital part	↘	–	↘
Right middle frontal gyrus orbital part	↘	–	–
Left inferior frontal gyrus, opercular part	↘	↗	–
Right inferior frontal gyrus, opercular part	↘	–	CD
Left inferior frontal gyrus, triangular part	↘	↗	–
Right inferior frontal gyrus, triangular part	↘	–	CD
Left inferior frontal gyrus, orbital part	↘	–	CD
Right inferior frontal gyrus, orbital part	↘	–	CD
Left rolandic operculum	↘	–	↘
Right rolandic operculum	↘	–	↘
Left supplementary motor area	↘	↗	CD
Right supplementary motor area	↘	–	CD
Left olfactory cortex	↘	–	↘CU
Right olfactory cortex	↘	–	↘
Left superior frontal gyrus, medial	↘	–	↘
Right superior frontal gyrus, medial	↘	–	↘ CD
Left superior frontal gyrus, medial orbital	↘	–	↘
Right superior frontal gyrus, medial orbital	↘	–	↘
Left gyrus rectus	↘	–	↘
Right gyrus rectus	↘	↘	↘
Left insula	↘	–	↘
Right insula	↘	–	↘
Left anterior cingulate and paracingulate gyri	↘	↗	–
Right anterior cingulate and paracingulate gyri	↘	–	↘
Left median cingulate and paracingulate gyri	↘	–	↘
Right median cingulate and paracingulate gyri	↘	–	–
Left posterior cingulate gyrus	↘	–	↘
Right posterior cingulate gyrus	↘	–	↘
Left parahippocampal gyrus	↘	↗	↗
Right parahippocampal gyrus	↘	–	–
Left calcarine fissure and surrounding cortex	↘	–	↘
Right calcarine fissure and surrounding cortex	↘	–	↘
Left cuneus	↘	–	↘
Right cuneus	↘	–	↘
Left lingual gyrus	↘	–	↘
Right lingual gyrus	↘	–	↘
Left superior occipital gyrus	↘	–	↘
Right superior occipital gyrus	↘	–	↘
Left middle occipital gyrus	↘	–	↘
Right middle occipital gyrus	↘	–	↘
Left inferior occipital gyrus	↘	–	↘
Right inferior occipital gyrus	↘	–	↘
Left fusiform gyrus	↘	–	–
Right fusiform gyrus	↘	–	–
Left postcentral gyrus	↘	–	↘ CD
Right postcentral gyrus	↘^a^F	–	↘
Left superior parietal gyrus	↘	–	↘ CD
Right superior parietal gyrus	↘	–	↘
Left inferior parietal, but supramarginal and angular gyri	↘	–	↘
Right inferior parietal, but supramarginal and angular gyri	↘	–	↘
Left supramarginal gyrus	↘	–	M ↘ CD; F ↘ CU
Right supramarginal gyrus	↘^a^F	–	↘
Left angular gyrus	↘	–	↘
Right angular gyrus	↘	–	CD
Left precuneus	↘	↘	↘
Right precuneus	↘	–	↘
Left paracentral lobule	↘	–	↘
Right paracentral lobule	↘	–	↘
Left heschl gyrus	↘	–	↘
Right heschl gyrus	↘	–	↘
Left superior temporal gyrus	↘	–	↘
Right superior temporal gyrus	↘	–	↘CD
Left temporal pole: superior temporal gyrus	–	–	↗CD
Right temporal pole: superior temporal gyrus	–	↗	↗CD
Left middle temporal gyrus	↘	–	CD
Right middle temporal gyrus	↘	–	↘CD
Left temporal pole: middle temporal gyrus	–	↗	↗CD
Right temporal pole: middle temporal gyrus	–	↗	↗CD
Left inferior temporal gyrus	↘	↗	CD
Right inferior temporal gyrus	↘	–	CD

CU, concaved up (U‐shaped trend); CD, concaved down (Inverted U‐shaped trend); –, no age effect; ↘, decrease with age; ↗, increase with age.

a *F*, sharper decrease in females than in males.

The relative volume of subcortical structures followed primarily a linear trend. However, while the hippocampi increased, the thalamus, caudate, and putamen decreased relative to the total cerebrum. Some of these observations are similar to those by Dennison et al. (2013), who found a linear volumetric increase in the hippocampi, along with a linear volumetric decrease in the caudate, putamen, and thalamus (Dennison et al. 2013). An earlier study by Muftuler et al. (2011) reported a contradicting observation of an age‐related relative volumetric increase in the thalamus; this could be due to the use of a narrower age range between 6 and 10 years and/or a different image processing software. Subfields of these subcortical structures were best described by either a linear or quadratic fit. Hippocampal CA1, cerebellar lobule VIIIB, and Crus I increased linearly, whereas lobules III, IV, and IX decreased linearly. Nonlinear trends include hippocampal CA2–3 and stratum radiatum (including the stratum lacunosum and moleculare), which followed inverted U‐shaped trends that peaked at around 12 years of age. It is interesting to note that an opposite trend of a U‐shaped trajectory that approached a minimum at 12 years of age was followed by cerebellar lobules VIIB and VIIIA. This reverse trend is a novel finding which suggests that different developmental patterns exist in the subcortex and have variable contributions to the volumetric changes during childhood and adolescence.

In all cases where a quadratic fit best illustrated the age‐related changes, a maximum or a minimum was attained at roughly 12 years of age. Cortical and subcortical developmental analyses are often decoupled because they appear to be seemingly unrelated (Shaw et al. 2008), but this similarity in developmental trajectories appears to imply similarity in periods of growth spurts and decline throughout the first two decades of postnatal life.

Sexual dimorphism also contributes to the developmental patterns of the subcortex. In many subcortical regions, females had a larger relative volume compared with males, independent of age. This was the case for the caudate, thalamus, right and left hippocampi, hippocampal CA4 (dentate gyrus), CA2–3, stratum radiatum, and cerebellar white matter. A similar observation of increased relative volume of the caudate and thalamus in females was made by Sowell et al. (2002). Giedd et al. (1997) also found a relative increase of the caudate, but with a relative decrease of the GP in females compared to males; the latter observation is inconsistent with our results. In several cerebellar lobules, girls actually had a smaller relative volume compared with boys, as was the case for lobules I–II, III, IV, and V. Interestingly, all of these cerebellar lobules are adjacent to one another and are positioned in the anterior lobe. These regions develop from a common portion of the rhombencephalon and have a similar timing of neurogenesis (Altman and Bayer 1997), suggesting that these cerebellar differences are caused by an underlying sex difference in developmental regulation of the cerebellum.

Additionally, in a few regions, the relative volume for the females was smaller in childhood but larger in adolescence compared to males, as was the case for the left cerebellum, the caudate, and the thalamus. To the best of our knowledge, while prior reports of sex‐by‐age interaction have been made, no similar observation on these specific structures has been published.

Since sex hormones are contributing to sexual dimorphism in brain development, longitudinally assessing changes as a function of sexual maturation instead of chronological age would allow a more accurate quantification of sex differences during adolescence. Goddings et al. (2014) carried out such investigation on selected subcortical regions and reported general developmental trends that resembled those reported here (e.g., an increase in relative hippocampal volume and a relative decrease in caudate and putamen volumes during development). Yet, they were also able to isolate more detailed sex‐by‐pubertal age interactions and developmental patterns, which are difficult to compare with cross‐sectional studies using only chronological age.

## Conclusion

This is the first comprehensive study that assesses not only cortical and subcortical, but also cerebellar growth trajectories and sexual dimorphism using carefully matched groups during normal development. Growth trajectories were found to follow primarily linear and quadratic trends. In the case of gray matter, the exact trend depended on the proportional contribution of the progressive changes, caused by synaptogenesis, and the regressive changes, caused by synaptic pruning, which varies across the brain. Sex differences were evident in the vertex‐based cortical thickness, the hippocampi, cerebellum, thalamus, and basal ganglia, indicating the role of sex on neuroanatomical structure throughout development.

## Conflict of Interest

None declared.
