# The developing human brain: age‐related changes in cortical, subcortical, and cerebellar anatomy

**DOI:** 10.1002/brb3.515

**Published:** 2016-06-14

**Authors:** Dafna Sussman, Rachel C. Leung, M. Mallar Chakravarty, Jason P. Lerch, Margot J. Taylor

In Sussman. D. 2016, “The” was inserted in the article title, so it reads as “The developing human brain: Age‐related changes in cortical, subcortical and cerebellar anatomy.”

The online version of the article has been corrected.
